# Meetings of Societies

**Published:** 1947

**Authors:** 


### Meetings of Societies

THE second meeting of the session was held on Wednesday, November 13th.

Dr. Milling was elected as *President Elect.* The election was received with considerable enthusiasm. Mr. Robert V. Cooke, Mr. Anthony Palin and Mr. Elwin Harris were elected to the Committee.

Mr. FitzGibbon then gave an account of **"Experiences with a Facio-maxillary Unit"** illustrated by a colour film ; shewing the really horrible effects produced by high explosives and the excellent repair that could be achieved by the elaborate and ingenious methods applied.

---

The third meeting was a clinical meeting at the Royal Infirmary on Wednesday, December 11th. A new procedure was adopted : six sets of cases were on view, and after this each set was demonstrated from the platform, and questions answered. Some of these cases are reported in this issue and we hope to publish the remainder.

---

The fourth meeting of the session was held on January 8th, 1947. Dr. J. R. Rees gave an address on **"Social Psychiatry and Medical Progress."** He pointed to the greatly increased interest in psychology during the last fifteen years ; and the importance of a correct psychological approach to the problems of physical disablement, particularly with reference to war and industrial casualties. He went on to ask that we should devote attention to the psychology not only of the individual but also to the group, especially that of the "special problem" group, illustrating his remarks by examples of "misfits" rescued by suitable reposting by the service psychiatric units. Occupational competence, he said, had much to do with morale and suggested that it would matter little to the nation if a large part of those who occupied hospital beds were allowed to die. For the sake of national survival it was of great importance that the nation as a whole should approach life's problems in an adult, balanced way : otherwise he foresaw further wars and the collapse of civilisations. It was the function of the Medical profession to lead the nation along the right path.

---

The fifth meeting of the session was held on Wednesday, February 12th. Professor H. J. Seddon, D.M., M.A., F.R.C.S., gave an account of **"The Epidemic of Anterior Poliomyelitis in the Island of Mauritius"** (*Lancet*, 1946, ii. 707).

---

The sixth meeting was held on Wednesday, March 12th, and devoted to three short papers. Mr. Watson-Williams : **"Penicillin in Ear, Nose and Throat"** ; Miss Beck : **"Head Injuries"** ; Dr. Sutton : **"Cardiac anomalies associated with Funnel-chest."**

